# GPER and Testicular Germ Cell Cancer

**DOI:** 10.3389/fendo.2020.600404

**Published:** 2021-01-26

**Authors:** Nicolas Chevalier, Charlotte Hinault, Stephan Clavel, Rachel Paul-Bellon, Patrick Fenichel

**Affiliations:** ^1^Université Côte d’Azur, CHU, INSERM U1065, C3M, Nice, France; ^2^Université Côte d’Azur, INSERM U1065, C3M, Nice, France

**Keywords:** testicular germ cell cancer, estrogen receptors, GPR30/GPER, endocrine disrupting compounds, fetal exposure, bisphenol A

## Abstract

The G protein-coupled estrogen receptor (GPER), also known as GPR30, is a widely conserved 7-transmembrane-domain protein which has been identified as a novel 17β-estradiol-binding protein that is structurally distinct from the classic oestrogen receptors (ERα and ERβ). There are still conflicting data regarding the exact role and the natural ligand of GPER/GPR30 in reproductive tracts as both male and female knock-out mice are fertile and have no abnormalities of reproductive organs. Testicular germ cell cancers (TGCCs) are the most common malignancy in young males and the most frequent cause of death from solid tumors in this age group. Clinical and experimental studies suggested that estrogens participate in the physiological and pathological control of male germ cell proliferation. In human seminoma cell line, while 17β-estradiol (E2) inhibits *in vitro* cell proliferation through an ERβ-dependent mechanism, an impermeable E2 conjugate (E2 coupled to BSA), *in vitro* cell proliferation is stimulated by activating ERK1/2 and protein kinase A through a membrane GPCR that we further identified as GPER/GPR30. The same effect was observed with low but environmentally relevant doses of BPA, an estrogenic endocrine disrupting compound. Furthermore, GPER/GPR30 is specifically overexpressed in seminomas but not in non-seminomas and this overexpression is correlated with an ERβ-downregulation. This GPER/GPR30 overexpression could be linked to some genetic variations, as single nucleotide polymorphisms, which was also reported in other hormone-dependent cancers. We will review here the implication of GPER/GPR30 in TGCCs pathophysiology and the arguments to consider GPER/GPR30 as a potential therapeutic target in humans.

## Introduction

Although relatively rare, testicular germ cell cancers (TGCC) are the most frequent solid cancer in young people (1, 2). Seminomas represent the most frequent histological form, occurring alone or associated with non-seminoma forms in 50-75% of cases (1, 2). Incidence rates of TGCC have been increasing worldwide for several decades (3, 4).

Risk factors for TGCC are described in **Table 1** and are mainly genetic. Indeed, incidence of TGCC is significantly increased in brothers and sons of TGCC patients (5, 7). Consistent with many epidemiological studies, gene variants that might predispose an individual to TGCC were identified by genome-wide association studies (GWAS) (8, 9). These variants included common variations on 12q22 in the KITLG gene, but also on PDE11A, BAK1, SPRY, DMRT1, DAZL, and PRDM14 [reviewed in (10)]. Other classical risk factors are cryptorchidism (or undescended testis), inguinal hernia, and all sexual differentiation disorders (6, 11) (**Table 1**).

**Table 1:** Usual risk factors of testicular germ cell cancers.

Risk Factor	Risk estimate or range Odd Ratio (95% CI)
Low birth weight (versus normal)	1.34 (1.08 – 1.67)
Low gestational age (versus not low)	1.31 (1.07 – 1.59)
Cryptorchidism	4.30 (3.62 – 5.11)
Inguinal hernia	1.63 (1.37 – 1.94)
Twinning	1.22 (1.03 – 1.44)
Prior TGCC	12.4 (11.0 – 13.9)
Father with TGCC	3.78 (1.94 – 6.63)
Brother with TGCC	12.74 (6.38 – 22.64)
Adult height (per 5 cm increase)	1.13 (1.07 – 1.19)

TGCC, testicular germ cell cancers.

Adapted from Cook MB. et al. (5). and Mc Glynn KA. et al. (6).

TGCC are considered to derive from a precursor lesion named “carcinoma *in situ* of the testis” or “germ cell neoplasia *in situ*” (GCNIS) (12). This lesion is present before birth, arising from the fetal germ cells (*i.e.* the gonocytes), and is reactivated after puberty under physiological hormonal stimulation (13). Epidemiological and clinical data have suggested that the increase of TGCC incidence could be related to environmental factors such as fetal exposure to endocrine disruptors (EDCs) with anti-androgenic and/or estrogenic effects (14, 15). However, this hypothesis supposes that TGCCs are estrogen-dependent tumors. In this review, we analyze the implication of classical and non-classical (GPER/GPR30) estrogen receptors in normal and malignant germ cells and the regulation of cell proliferation by xeno-estrogens and discuss how GPER/GPR30 could be considered as a potential therapeutic target in humans.

## Could TGCC Be a Hormone-Dependent Cancer?

### Environmental Features

Several studies have reported abnormalities of male genital tracts in animals that were accidently exposed to endocrine disruptors, such as hypospadias and cryptorchidism in alligators (16) or panthers (17), especially in the case of exposition to the organochloride dichlorodiphenyltrichloroethane (DDT) or its metabolites (DDE, DDD), which exhibit estrogenic properties. However, there is actually no animal model of TGCC, except for transgenic mice with targeted overexpression of GDNF in spermatogonia (18).

In humans, early fetal exposure to diethylstilbestrol (DES), a synthetic estrogen used during the 1960’s, was responsible for an increased incidence rate of cryptorchidism and hypofertility by impairment of sperm quality in sons and in grandsons (19, 20). Such an exposure was also suggested to be responsible for the occurrence of TGCC in the offspring of two meta-analysis (21, 22). In past studies, the association between occupational exposure and risk to develop TGCC (23–25) was well-documented and offered suggestive or strong arguments. However, more recent epidemiological case-control studies reported conflicting data for fetal exposure to p,p′-DDT (estrogenic compound) or to p,p′-DDE (a stable metabolite of DDT with antiandrogenic properties) (26–31).

### Estrogens and Normal Germ Cells

Testicular concentrations of 17β-estradiol (E2) are 10 to 100 times higher than those measured in blood (32). E2 is produced after testosterone conversion by aromatase in all mammalian testes, including humans (33). Estrogens are essential for spermatogenesis control but the type of estrogen receptors involved and the molecular mechanisms by which estrogens may precisely act during spermatogenesis still remain incompletely understood (34).

Expression of classical and non-classical estrogen receptors expression in mammalian testes is well-established. It exhibits some species specificity and some controversial results, especially in humans [reviewed in (35)]. Indeed, in humans, the classical nuclear estrogen receptor ERβ has been clearly identified in most germ cells, including fetal gonocytes (36), neonatal, prepubertal (37), and adult spermatogonia (38), while ERα is not expressed in human gonocytes (36) or neonatal or prepubertal spermatogonia (37). However, data concerning the expression of ERα by male germ cells are inconsistent, as some authors reported an expression in elongated spermatids and mature spermatozoa (39) and others did not find any expression of ERα at all (38, 40). In fact, these inconsistent observations could be due to the existence of a truncated isoform of ERα lacking exon 1, called ERα46, which has been identified in human adult spermatozoa (41). This isoform could participate in non-genomic membrane signaling. Indeed, one reported case of a man with an inactivating mutation of ERα gene was associated with a normal sperm count but with completely abnormal motility (42).

### GPER/GPR30 and Testis

GPR30 is a widely conserved orphan GPCR, which has been renamed as G protein-coupled estrogen receptor (GPER) (HUGO & MGI Databases). It is a seven-transmembrane domain protein, identified for the first time in a triple-negative breast cancer cell line, that can bind E2 and other estrogenic compounds independently of the classic estrogen receptors (ERα and ERβ). The precise subcellular localization of GPER/GPR30 is still a matter of debate as it has been detected at the plasma membrane but also in the endoplasmic reticulum and Golgi apparatus (43).

GPER/GPR30 has been identified in numerous rodents and human estrogen targets normal or malignant tissues where it can mediate rapid E2-induced non genomic signaling events (43). GPER/GPR30 can activate cell proliferation through several signaling pathways involving MAP kinases, ERK1/2, and PI3K pathways (44, 45) but also microRNA regulation (46–48), EGFR transactivation (49, 50), HIF induced pathway (51, 52), IGF-R pathway (53, 54), NF-kB pathway (55, 56), and crosstalk with other receptors (classical or truncated estrogen receptors, or other steroids receptors) (57–59). Within those pathways, the activation of ERK1/2 is undoubtedly the most consistent pathway across cell types and is usually considered as a key factor in cancer prognosis.

Analyzing normal human testes from a fertile man, we previously reported that GPER/GPR30 was expressed by both somatic (Sertoli and Leydig cells) and germ cells (60). Amazingly, Rago et al. (61). reported a negative staining in adult germ cells, probably due to the use of abnormal granulomatous testes. As expression of GPER/GPR30 in human fetal gonocytes has not yet been studied; it could be possible that only immature germ cells and gonocytes express GPER/GPR30, explaining these inconsistent data [reviewed in (62)].

## Estrogens, Germ Cells Proliferation, and TGCC

### Estrogen Receptors and Malignant Germ Cells

Estrogen receptor expression is a well-recognized prognosis factor of estrogen-dependent cancers, especially in the case of breast cancer (63–65). Several teams have suggested that TGCCs could be estrogen-dependent cancers as they express both ERβ and GPER/GPR30 (66–70). We previously reported in a large cohort of TGCCs that GPER/GPR30 was overexpressed only in seminoma but not in non-seminoma tumors (60) and promoted seminoma cell proliferation (71). Pais et al. (72) reported that expression of ERβ was decreased in seminoma but remained high in teratomas. In the same way, Boscia et al. (69) showed that ERβ was downregulated in seminomas and reported a negative association between the expression of ERβ and GPER/GPR30 protein. This inverse receptor expression pattern could reflect a switch in estrogen responsiveness from a suppressive (66) to a promoting profile (60, 67), as it has also been observed in other estrogen-dependent cancers and was correlated to a poorer prognosis (63–65).

Genetic factors could of course explain this specific profile of expression. Variants of ERβ were explored but studies reported inconsistent data. Ferlin et al. (73) reported a weak but not significant association between one variant for ERβ and an increase risk of TGCC in Italian men, while Brokken et al. (74) described exactly the opposite in a cohort of 367 Nordic patients with TGCC and two other variants of the ERβ. In our large cohort of 169 TGCCs, we were able to describe that seminomas were characterized by a loss of homozygous ancestral genotype concerning two polymorphisms located in the promoter region of GPER/GPR30 (75). We assumed that this genotype could explain a part of GPER/GPR30 overexpression in seminomas. This expression profile could also be determined by epigenetic modulation of ERβ and GPER/GPR30 genes (low expression of ERβ due to an hypermethylation of its promoter and high expression of GPER/GPR30 gene due to an hypomethylation of its promoter). Indeed, fetal exposure to EDCs is supposed to induce such epigenetic modulation as reported, for example, by Zama et al. (76) who reported that fetal and neonatal exposure to the endocrine disruptor methoxychlor was responsible for a down regulation of ovarian ERβ gene expression.

Anway et al. (77) were the first to observe and to report several epigenetic modifications in rodent DNA male germ cells after gestational exposure to vinclozolin (antiandrogenic compound) or methoxychlor (estrogenic compound). These data have been recently confirmed by Dumasia et al. (78) for xenoestrogens signaling through ERβ. Since this first publication of Anway et al. (77) DNA methylation (hyper- and hypo-) (79, 80), onco-miRNAs expression (miR 371-373) (81, 82), or chromatin modifications have been reported in TGCC (83). However, even if experimental data in rodents suggested that these epigenetic modifications might be induced by fetal exposure to EDCs, it remains to be proven that such epigenetic modifications exist in humans and can be induced by fetal exposure to EDCs.

### Putative Role of GPER/GPR30 in Malignant Germ Cells

JKT-1 cell line is derived from a human testicular seminoma (84), which expressed functional aromatase (66) and is able to convert testosterone into E2 and as well as ERβ, but not ERα. At physiological concentrations (10^-7^ to 10^-9^ M), we previously reported that E2 was able to inhibit *in vitro* JKT-1 cell proliferation involving an ERβ pathway (66). We conjugated E2 to bovine serum albumin (E2-BSA) for the purpose that E2 cannot cross the plasma membrane and then cannot link to its canonical receptor ERβ. In this condition, E2-BSA at the same concentrations (10^-7^ to 10^-9^ M) stimulated *in vitro* JKT-1 cell proliferation by activating the ERK1/2 and PKA pathways. E2-BSA is responsible for a rapid (15 min) phosphorylation of CREB. This effect was not inhibited by ICI-182,780, an antagonist of ERβ, but by *Pertussis toxin*, suggestive of the involvement of a membrane G-protein-coupled receptor (GPCR). Similar results were obtained with bisphenol A (BPA) at low and very low (nM to pM) concentrations (85), the levels already found in male cord blood and in more than 95% of the worldwide population (86, 87).

Among EDCs, BPA is especially a matter of concern as populations exhibit worldwide with detectable blood and/or urine levels of BPA (86), and so it is used as a monomer to manufacture a wide range of objects containing polycarbonate plastic and resins. BPA is considered an estrogenic EDC and is recognized as a substance of very high concern (SVHC) by the European Chemicals Agency (ECHA) because several experimentations and data reported that it is involved in developmental, reproductive, and malignant diseases by mimicking the natural hormone E2 and by interfering with endogenous pathways at selective periods, especially during fetal life (88). However, BPA exhibits a weak affinity for the classical ERs, which is 1,000–2,000 times lower than E2. Thus, it has been suggested that BPA could act through other receptors than classical ERs, for example GPER/GPR30, PPARγ gamma, or ERRγ gamma (88).

**Figure 1:**
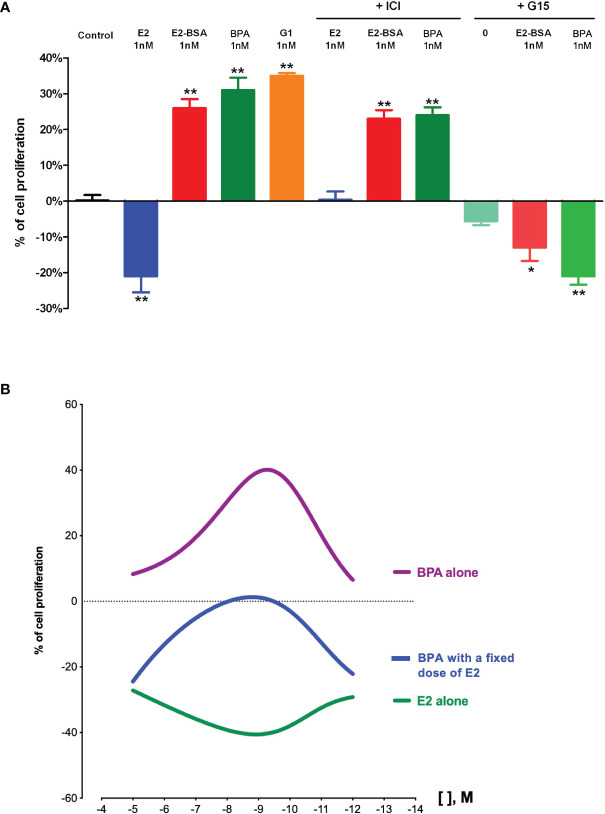
Effects of estrogens and bisphenol A on human testicular seminoma cell (JKT-1) proliferation *in vitro*. **(A)** Analysis of JKT-1 cells proliferation *in vitro*, adapted from Chevalier et al. (71) JKT-1 cells were seeded in six-well plates (0.6 × 10^6^ cells/well). After 48 h, the JKT-1 cells were washed and estrogen starved overnight in phenol red-free DMEM (Dulbeccos’s Modified Eagle Medium) supplemented with 1% charcoal-stripped fetal bovine serum. Serum-deprived JKT-1 cells were then incubated for 24 hours with 17β-estradiol (E2; 1 nM), E2-BSA (1 nM), or bisphenol A (BPA; 1 nM), after a pre-treatment with G15 (1 nM) or ICI-182,780 (1 µM). G1 (1 nM) was used as a positive control. Values shown are expressed in percent change in cell number compared to control (steroid-free medium containing DMSO for bisphenol A or medium containing ethanol for estrogens, G1, and G15) given as the mean ± SE of at least three independent experiments. Cell counting was performed using a Malassez hemocytometer and confirmed using Vi-CELL automate (Beckman Coulter, Fullerton, CA). *p < 0.05; **p < 0.001. **(B)** Dose-response curves obtained with 17β-estradiol (E2) and bisphenol A (BPA) in JKT-1 cells *in vitro*, adapted from Fenichel et al. (89). and Bouskine et al. (85). JKT-1 cells were seeded in six-well plates (0.6 × 10^6^ cells/well). After 48 h, the JKT-1 cells were washed and estrogen starved overnight in phenol red-free DMEM (Dulbeccos’s Modified Eagle Medium) supplemented with 1% charcoal-stripped fetal bovine serum. Serum-deprived JKT-1 cells were then incubated for 24 hours with 17β-estradiol (E2) alone or bisphenol A (BPA) alone at variable doses from 10^-5^ M to 10^-12^ M obtained by serial dilutions, or with a fixed dose of E2 (10^-9^ M) and BPA at variable doses (same range, from 10^-5^ M to 10^-12^ M). Values shown are expressed in percent change in cell number compared to control (steroid-free medium containing DMSO for bisphenol A or medium containing ethanol for estrogens) given as the mean ± SE of nine independent experiments for each condition. Cell counting was performed using a Malassez hemocytometer and confirmed using Vi-CELL automate (Beckman Coulter, Fullerton, CA). Modeling of dose-response curves were performed using GraphPad Prism version 8.4.3 for Mac OS X, GraphPad Software, San Diego, California USA, www.graphpad.com.

In our JKT-1 seminoma cells model, we were able to identify the GPCR involved in the promoting action of E2-BSA and BPA as GPER/GPR30 (71). Indeed, the BPA-induced promotive effect was mimicked by G1 alone, a specific agonist of GPER/GPR30, while it was totally inhibited by G15, a partial antagonist of GPER/GPR30, as well as a selective anti-GPER/GPR30 siRNA (**Figure 1A**) (60, 71). This GPER/GPR30-mediated signaling of BPA was also reported in other hormone-dependent tumors. For example, Pupo et al. (90) reported that BPA could increase the proliferation of SKBr3 breast cancer cells, which lack the classical ERs, through a GPER/GPR30-EGFR/ERK transduction pathway.

Interestingly, the dose-response curve that we obtained for BPA in our model was non-monotonic and showed an inverted U-shape curve (**Figure 1B**). Non-monotonic dose response curves (NMDRC) have already been reported and well-documented for natural hormones. NMDRC have also been suggested for EDCs, especially in the case of BPA, but there are few consistent data available in the literature (91). Most authors explained that these atypical dose-response curves resulted from the complex interactions between the ligand (*i.e.*, the natural hormone or an EDC) and a hormone receptor. In our model, it could, for example, be explained by the resultant of the double opposite effect of BPA on ERβ and GPER/GPR30 (60, 85). Indeed, at low doses (nM or pM), BPA acts only through GPER/GPR30 by a promotive effect while it acts also through ERβ at higher dose (mM), which counteracted the promotive GPER/GPR30-mediated effect (66). In order to confirm this hypothesis, we exposed JKT-1 cells to variable doses of BPA together with a fixed dose of E2. The BPA dose-response curve that we obtained kept its inverted U-shape aspect but was down-translated, confirming that BPA can act either through ERβ or GPER/GPR30 depending on the other estrogenic compounds that are present in the cell environment. This parameter is particularly important to consider since in most cases we are exposed to EDC mixtures.

Furthermore, in the same cellular model, the effects of several EDCs on *in vitro* proliferation were totally different and dependent on the resultant of the two expressed receptors, ERβ and GPER/GPR30. For example, atrazine, another estrogenic pesticide, induced a suppressive effect on seminoma cell proliferation *in vitro* involving a GPER/GPR30-dependent pathway (92). In the same way, an alkylphenol mix promoted seminoma cell proliferation through a GPER/GPR30-dependent pathway (93). However, in this case, the promoting effect is also mediated through ERα36, which is a truncated form of the canonical ERα66 (without both transcriptional activation domains (AF-1 and AF-2)) and was first described first by Wang et al. (94) in 2005. It seems to participate in non-genomic estrogen signaling concurrently to and/or associated with GPER/GPR30, as demonstrated in breast cancer cell lines (94) and in seminoma-like TCam-2 cell line (95). Thus, the presence of ERα36 in tumors is an important parameter to consider before considering selective antagonists of GPER/GPR30 as a therapeutic target in TGCC or other estrogen-dependent cancers.

The crosstalk among GPER/GPR30 signaling, classical estrogen receptors, and other nuclear receptors involved in testis physiology regulation is also important to consider (96). Through such interactions, GPER/GPR30 could probably modulate the tumor microenvironment and through this mediate TGCC progression and aggressiveness, especially by inducing epithelial-mesenchymal-transition (97, 98), as has been reported in breast cancer (98, 99) and in pancreatic adenocarcinoma (100).

## Could GPER/GPR30 Constitute a Potential Therapeutic Target for TGCC?

Accumulating evidence supports the role of GPER/GPR30 in cancer progression and metastasis in estrogen-dependent cancers (especially in breast cancer), even though GPER/GPR30 signaling can differently affect the development of cancer depending on the type of tissue, but also in the same tissue depending on the type of ligand (92). A better comprehension of the molecular pathways involved in TGCC development, in particular the role of GPER/GPR30 in tumor progression, points out new tools like agonists or antagonists of GPER/GPR30, which could be used going forward by clinicians to target cancer cells and improve the patient’s chance of survival (68, 101).

Three pharmacological GPER/GPR30-ligands were routinely available to study GPER/GPR30 functions. The first one, G-1, was identified by Bologa in 2006 and is a specific agonist of GPER/GPR30, while G-15 and G-36, identified respectively in 2009 and 2011 by Dennis, are GPER/GPR30 antagonists. However, G-15 exhibits a partial cross-reactivity with ERα explaining why G-36 is mainly used in the study of GPER/GPR30 (102). Other pharmacological ligands were synthetized (GPER/GPR30-L1 and GPER/GPR30-L2) (102, 103) but they exhibit variable affinities for GPER/GPR30 and potential cross-reactivity with classical ERs, explaining why they cannot be considered as therapeutic tools at this time (104). These small molecules were used especially *in vitro*, as we did with seminoma cells; in our model, G-1 was able to mimic the proliferative effect of BPA while G-15 neutralized this effect and reduced cell proliferation in the presence of BPA (71). Thus, G-15 may be a helpful adjuvant in the treatment of TGCC. Nevertheless, to date, no studies have reported the use of GPER/GPR30 antagonists in this way.

However, agonists and antagonists of GPER/GPR30 were tested in the treatment of other tumors. For example, as we observed *in vitro* in seminoma cells, G-15 was also able to decrease the *in vitro* proliferation of non-small cell lung cancer (105) while G-1 was reported to induce malignant cell proliferation, invasion, and migration in primary cultured lung cancer cells (106) and in ER-negative breast cancer cells (107, 108) involving SIRT1 (108). At the opposite end, G-1 was able to decrease *in vivo* the tumor volume of pancreatic ductal adenocarcinoma in mice (109) and of adrenocortical carcinoma in a xenograft model (110, 111).

Interestingly, G-1 was also able to reduce the side effects of chemotherapy, as, for example, the cardiac toxicity of doxorubicin is usually used as an adjuvant therapy in breast cancer (112). This beneficial effect is related to the well-documented GPER/GPR30 actions on the vascular system, involving in this specific case the Nox1 pathway, which could constitute new therapeutic tools (113, 114).

Actually, only one clinical study is registered in Clinical Trials involving a GPER/GPR30 agonist. The NCT04130516 is a phase 1, first-in-human, open-label, multicenter study (up to six study sites in the United States) designed to characterize the safety, tolerability, and antitumor effects of LNS8801 administered orally in patients with advanced cancer (solid tumor or lymphoma). The recruitment is still on-going, and the estimated primary completion date is the end of 2021.

Finally, even though GPER/GPR30 modulation represents a potential novel strategy in cancer therapy, there remains a lack of solid clinical evidence supporting the specificity of GPER/GPR30 antagonists, especially in TGCC.

When compared with normal tissues, GPER/GPR30 is highly expressed in breast cancer and its high expression at the plasma membrane is strongly correlated with a poor prognosis, especially in triple negative tumors (115). This overexpression of GPER/GPR30 was also related to tamoxifen resistance (116, 117). Thus, GPER/GPR30 could be considered as a potential therapeutic target in such estrogen-dependent cancers.

## Conclusion

Since its discovery in breast cancer, the role of GPER/GPR30 in estrogen-dependent malignancies has been of great interest. TGCC, the most common solid cancer in young men, expresses classical estrogen receptors (ERβ) but also GPER/GPR30. While E2 is responsible for a suppressive effect through an ERβ-dependent pathway, EDCs like BPA could induce *in vitro* seminoma cell proliferation by binding to GPER/GPR30. Furthermore, GPER/GPR30 is overexpressed in seminoma, probably due to genetic and/or epigenetic modulations that could be induced by fetal exposure to some EDCs. As proposed by Skakkebaek (4), an estrogenic environment might impair normal differentiation and proliferation of normal fetal, perinatal, and peripubertal germ stem cells, and then predispose an individual to TGCC, meaning it may be considered as an estrogen-dependent cancer. In our model, we have showed that G-15, a partial antagonist of GPER/GPR30, was able to reduce *in vitro* the BPA-induced cell proliferation (71) and may constitute a potential adjuvant in the treatment of TGCC. However, there remains a lack of solid clinical evidence to consider its clinical use. Direct regulation of GPER/GPR30 expression by siRNA silencing and/or nanotechnology could offer, at last, another tool to target GPER/GPR30 in cancer therapy.

## Author Contributions

NC and PF designed the study and contributed to the discussions and manuscript. NC and RP-B researched and interpreted data. SC and CH contributed to discussions and manuscript. All authors contributed to the article and approved the submitted version.

## Funding

This work was supported by the Société Française d’Endocrinologie (SFE), the Institut National de la Santé et de la Recherche (INSERM), and the Fondation pour la Recherche Médicale (FRM) (to NC).

## Conflict of Interest

The authors declare that the research was conducted in the absence of any commercial or financial relationships that could be construed as a potential conflict of interest.
